# Life experiences leading to the choice of surgery—A qualitative study exploring reasons behind the choice of undergoing gender affirmative surgery

**DOI:** 10.3389/fsoc.2023.1226959

**Published:** 2023-09-14

**Authors:** Lene Kjelkenes Bjørnson, Mette Sagbakken

**Affiliations:** ^1^Department of Gender Identity Assessment, Norwegian Centre for Gender Incongruence, Oslo University Hospital, Oslo, Norway; ^2^Faculty of Health Sciences, Department of Nursing and Health Promotion, Oslo Metropolitan University, Oslo, Norway

**Keywords:** gender dysphoria, gender identity, gender affirmative care, gender affirmative surgery, transgender

## Abstract

**Objective:**

Gender dysphoria is frequently accompanied by physical dissatisfaction and body image issues. The primary objective of this study is to explore subjective experiences and perceptions among those who has undergone gender reassignment surgery, as well as their retrospective path to that decision.

**Method:**

Sixteen qualitative in-depth interviews were conducted with 9 participants. The participants were accepted for gender affirming surgery and interviewed before and after surgery.

**Results:**

Cultural norms, and values in relation to time and context were highlighted as significant in reference to the opportunity one had to display a gender identity that corresponded to prevailing expectations. Participants gradually began to recognize their differences and divergence from others in social interactions and experiencing “wrong” bodily changes during puberty created even greater discrepancy. Several impression control measures, such as avoiding certain situations and using concealing techniques, were employed to prevent what was described as both felt and enacted stigma. The significance of having genital organs that accurately reflect one's gender identity was emphasized to prevent emotional distress and dysphoria caused by this discrepancy.

**Conclusion:**

Socio-cultural expectations, combined with enacted stigma, seem to cause, or re-enforce self-stigma as people internalize these attitudes and suffer from physical and mental consequences as a result. Thus, societal, and cultural trends seem to have a strong influence and feed the idea of being born in the wrong body. However, even though several participants underwent socially inspired alterations, they all experienced dysphoria in the extent that they continued to see reassignment surgery as a solution.

## Background

Transgender is an overarching term for individuals who identify with a gender that are inconsistent or not culturally associated with their assigned sex (van de Grift et al., 2016). The complex phenomenon of gender dysphoria (GD) is described in the fifth edition of the Diagnostic and Statistical Manual of Mental Health Disorders (DSM-5, American Psychiatric Association, 2013). According to the World Health Organization (WHO) classification of diseases, the diagnoses are gender dysphoria (GD) or gender incongruence (World Health Organization, 2019). GD is a condition in which a person is distressed due to a mismatch between their assigned and experienced gender. The origin of such mismatch is debated and can be interpreted, partly or fully, as a product of normative cisgender expectations (Williams, 2014). Dysphoria and gender incongruence are symptoms that can develop in infancy, adolescence, or in adulthood, and are conditions in which a person's gender identity and gender at birth are at odds. Gender dysphoric patients are often characterized by the pursuit of gender affirmative treatment, which include feminization and masculinization of the body through hormone therapy and surgery (Nieder et al., 2011; Gulbrandsen, 2019).

According to the criterion, Z76.80, feelings of hatred and inadequacy for one's own physical sex are commonly associated with a strong wish to live and be accepted as belonging to the other sex. Thus, these emotions frequently result in the desire for surgery or hormonal treatment to as nearly as possible alter the physique to match the chosen gender. In Norway, where the current study is conducted, these bodily experiences or emotions must have lasted at least 2 years and not be an indication of any other mental disorder such as schizophrenia, or genetic or chromosomal abnormalities (Gulbrandsen, 2019).

### Empirical background

In the 1990s, transgender theory and research began to emerge in the United States. The first definitive expression of the field is said to be Stones and Stryker (Stryker and Whittle, 2013), who gathered fifty influential texts with new introductions by the editors that, taken together, documented the evolution of transgender studies in the English-speaking world. Several scientific fields have contributed to the complex and interdisciplinary topic of transgender research. Research on hormonal and surgical treatment has been conducted both nationally and internationally (Tønseth et al., 2010; Bouman and Arcelus, 2017), and transgender research has increased in recent years (Galambos, 2004; Eliason et al., 2010; Kreukles et al., 2010; Fausto-Sterling, 2012). The quality of the studies varies due to a variety of factors, including the difficulty in locating a representative population and the scarcity of randomized controlled studies (Heylens et al., 2014). Most of the available research has focused on transgender health and quality of life (Dhejne et al., 2011), health service interaction, and surgical intervention outcomes (Tønseth et al., 2010).

When this study was initiated in 2019, there were few studies on the subjective experiences of transgender persons. Sociologists Hines (2007) and Ekins and King (2002), gender researcher Stryker and Whittle (2013), as well as sexologists Bouman and Arcelus (2017), are acknowledged as key contributors in international studies and literature. Van der Ros (2013), a gender researcher, has contributed to raising awareness about the challenges faced by transgender people, including subjective aspects. They all stress the complexities of gender in a way that makes it understandable to everyone, not just academics and health-care specialists. Benestad, a sexologist and physician, as well as Almås, a psychologist and sexologist, have contributed to the understanding of the transgender phenomena in Norway (Benestad, 2010; Almås and Benestad, 2017; Benestad et al., 2017). They emphasize the importance of understanding gender variance, and that shame is a function of how gender and transgender identities are formed, recognized, accepted, and validated in different socio-cultural contexts.

The meaning and relevance of shame among transgender people are discussed in Giordano's (2018) literature study of gender, culture, and shame from the 1900s to 2017. The findings demonstrated that the therapeutic influence of shame cannot be overestimated, given the substantial risks associated with it, and the paper shed light on the link between shame and self-destruction. The author claims that shame is significant since it exists outside of the intellect, and Giordano also contends that shame has a social basis, and that shame is a function of how gender and transgender identities are formed, acknowledged, or rather vexed in certain social-cultural settings (Giordano, 2018).

There are some new studies concentrating on transgender people's subjective experiences of living as their assigned gender before treatment and surgery. Jessen et al. (2021) investigated the subjective experiences of GD among help-seeking transgender people assigned female at birth. They found that a variety of physical signs in the body throughout the day triggered GD, as well as emotional recollections of being different. Social interaction proved to be crucial since it affected how one's subjective experiences of GD changed based on the situation and how others responded to them. The participants told how the process of “coming out” was a transformative experience that changed how the participants understood themselves and previous experiences. However, everyday life required careful negotiation to feel “whole,” and these efforts came at a price, as some participants tended to dislike their body even more and developed new forms of GD as a consequence of committing to the male identity. Based on the results, the authors suggest a more conceptually nuanced model of GD, where bodily sensations and emotional memories from the past are sources that elicited GD.

Elian Jentoft's master thesis (2019) investigated the experiences of young individuals with gender incongruence and their families in Norway. The findings show that the study participants experienced puberty as a “crisis” which made them feel that they needed immediate preventative interventions. Further, the thesis showed how these pubertal experiences informed conceptualizations of gender affirming care (GAC) as a “lifesaving” treatment. Another Norwegian study sought to gain a deeper understanding of the experience of transgender women in terms of identity and self-understanding (Moen and Aune, 2018). The self-understanding of the participants found different expressions, depending on personality and life experiences, and whether one had “come out of the closet.” The participants who had chosen to openly express their perceived identity seemed to feel more secure than those who had not. However, the feeling of belonging and recognition was highly influential and some preferred to live as males out of consideration for those in their surroundings. The study conclude that gender identity may primarily be a cognitive and emotional condition and not necessarily a feeling of being born or confined into to ‘the wrong body'. Another qualitative master thesis form Norway (Lerfaldet, 2019) also emphasizes the significance of the social environment regarding the participants prospects of living as their perceived gender. The study found that both intimate relationships, relationships within one's social circle, and the relationship between the participant and the existing norms of the society all can contribute positively and negatively to one's self-esteem and the perceived possibility to live and express themselves as they want.

Two further Norwegian studies explored the relationship between transgender people's sexuality and their bodies. Bolsø (2019) explored the tension between the physical body and the visualization of the body in transgender persons seeking medical correction of the body. The results indicated an underlying drive to remove bodily parts due to the strong symbolism of the present genitals, representing sources of mental unrest and disturbance. Another finding was that persons who identify as transgender experienced hormone-stimulated ambivalence, as the sudden and severe alterations in body image brought on by hormonal therapy, caused new and acute conditions both in relation to symbolic decoding and the actual use of the genitals during sexual activity. The authors argue that there is not principal difference between transgender people and others when it comes to possible- and potentially changing tensions between the ideas about own body and the actual body.

Although transgender people's understanding of themselves and their identity has been described in literature and empirical studies their subjective reasons to undergo medical and surgically treatment, particularly genital surgery, has received little attention in research (Moen and Aune, 2018).

### Aim

This study is part of a larger research project aiming to learn more about GD and factors leading to medical and surgical intervention. Thus, we aimed to explore the variety of subjective experiences and interrelated reasons why transgender people undergo bodily modifications. A greater comprehension of subjective experiences of persons with GD may enable us to better comprehend and treat the individual.

### Theoretical framework

Parts of the analysis will draw on Goffmann's (1992) interaction and identity-building theory to show how people in society perform or act out their gender roles dependent on their gender identification. Goffmann's (1992) theory is marked by a dramaturgical approach; Goffman being inspired by the theatrical scene in developing the theory. The role theory is concerned with interpersonal interaction, and the first impression is central for how the interaction will take place as all participants will form expectations and execute performances related to the roles they will be expected to play. Goffmann (1992) also discusses how an individual may position herself/himself toward the other participants because of his/her active engagement in the interaction, and therefore take on a role that will influence how the others connect to him or her. Goffmann (1992) distinguishes between “back-stage” and “front-stage” in this interaction. One part is performed out “frontstage,” whereas backstage is where the “real” role development takes place, and where the individual plans how he or she will appear in the role. While Goffmann (1992) claims that individuals play roles for a variety of reasons, he also contends that certain people will seek out positions that guarantee their acceptability and acknowledgment by others to avoid both felt and enacted stigma.

## Methods

In this part of the study, we sought to explore the subjective experiences of transgender people who are undergoing bodily modifications and the study was guided by the following research question: *Which life experiences, impressions, and ideas influenced individuals' decisions to get gender-confirming surgery?*

### Study design

Narrative in-depth interviews were regarded as the best method for exploring unique life experiences. The primary objective of this part of the study was to investigate the participants' subjective experiences and ideas and how those lead to treatment seeking. However, as part of the overall study we also sought to explore experiences related to interactions with the clinic before and after the genital surgery; thus, the interviews were done both before and after the surgery. This part of the study will contain results from the pre-surgery interviews, analyzed with a focus on the research question above.

### Study setting and participants

The study participants were all diagnosed with Z.76.80, had started hormonal treatment, and were undergoing surgery at Oslo University Hospital (OUS). Since 1979, OUS has had a country function for the treatment of individuals diagnosed with Z.76.80 (F-64.0), gender incongruence (ICD-10), the current indication position for gender-affirming medical and surgical procedures (Gulbrandsen, 2019).

The participants were recruited by an experienced patient coordinator in one of OUS's departments. The participants come from all around Norway, as the current department is the only one that does gender affirming genital surgery. Ten people were recruited to be interviewed before and after the gender affirmative surgery. After the interviews (one pre- and one post-surgical), one person withdrew consent, and two individuals were unable to attend the follow-up interview owing to a continuing pandemic. Thus, the study includes nine in-depth interviews before surgery and seven in-depth interviews after surgery. In this part of the study the 9 in dept-interviews before surgery is the basis for the analysis.

### Data production

The interviews took place between August 2018 and December 2020. The interviews were conducted and transcribed by the first author. To facilitate transcription and contextualize the interviews, field notes were kept with each one. All interviews were tape-recorded, and the pre-surgery interviews, took place 2 weeks before the surgery. The interviews lasted between 45 min and 1 h and 15 min and took place in a consultation room at the hospital. Because of the narrative interview structure, the interviews were conducted in an open manner, and all patients received the same three questions from the interview guide; (1) *Can you tell me a little about your background?* (including probes), (2) *Can you tell me a little about what your expectations are?* (in front of surgery), and (3) *Can you tell me about the process leading to the decision to undergo genital surgery?* The follow-up questions were all unique.

### Analysis

We used thematic analysis to identify patterns within the material and followed the guidelines for thematic analysis outlined by Braun and Clarke (2006). Conceptual maps and colors were used to visualize associations between different categories that emerged from the material, and the notes taken during the interviews represented additional analytical sources. All transcriptions were manually coded within a coding frame based on Braun and Clarke (2006). The analysis followed these steps: (1) reading all the material to get an overall impression; (2) identifying units of meaning that represent the individual experience and coding for these; (3) condensing and summarizing the content of each of the coded groups; and (4) integrating the insights from the condensed meaning units into generalized descriptions that reflected apparently significant factors. The analysis was summarized and accounted for in an analysis document that were discussed and negotiated with the co-author.

### Ethics

A formal request for participation was sent out in advance, and all participants signed a consent form. The individuals are described using fictional names to ensure de-identification and confidentiality. Norwegian center for research data (NSD) approved the current study. The project was evaluated by the regional committee for medical and health research ethics (REK), which gave its prior approval (2019/731 B).

## Results

### Deviating from established norms and values

Cultural norms, and values in relation to both time and context were highlighted as significant in reference to the opportunity one had in relation to expressing a gender identity that corresponded to expectations. Values and norms that, for example, applied when the participants were young could be very different from those that pertained when they became adults. Ella, 23 years old, describes this discrepancy:

The year I was born, you could practically say that girls should have skirts and boys should have jeans, and girls should have long hair and boys should have short hair. […] The joke my dad always made; it was such a joke that you make to the boys all the time. ‘Go and cut your hair and get a job.' It was something I heard when I was younger, and it was something that could make me sad.

Appearance, clothing style and hairstyle are for many described as something they were classified by as children and adolescents. Aleksander 21 years talked about how he tried to meet the expectations of gratefulness and joy when receiving his (female) national costume as a confirmation gift:

I got a “bunad” (national costume) from my grandmother as a confirmation gift, and it was great ... But, to see that expensive garment, tremendously expensive garment […] … and I was just looking forward to taking it off, and after I have not really put on the bunad once. So, you kind of lie a bit just to meet the requirements of those around you.

Cross-cultural differences in gender expressions and behavioral expectations were also addressed as a complicating factor. Ingrid, 36, born in Asia, experienced that due to the context she was raised in, it was even more difficult to deviate from what was expected, and her parents were openly criticized: “*My parents' friends said they had to teach me, they said to my parents that they must teach me to be a boy, such a real boy again. I could not be in the middle…*.”

Being different and her parents' inability to manage it also led to violent incidents, committed by both family and friends: “*When I was in my home country, I was also beaten and harassed. Not just me, but also my family*.”

Regardless of context and in a variety of ways, all participants shared narratives that described experiences of feeling different in a way that had social ramifications. Maria, a 21-year-old, explains how feeling self-conscious, but also anxious about her identity made her isolated:

There were a lot of people (parents) who thought I was going to make their children abnormal and make their children no longer children because I was so incredibly grown up, and I had worries and thoughts that no one else had. And then in the end other children were told to stay away from me.

Several participants told how they were aware of their parents' or others' annoyance or expressions of judgment about what they were doing or how they seemed. Many stated that they wanted to live up to the demands around them but that they often failed to do so. Not feeling accepted or included often led to feelings of shame, both of themselves and on behalf of the family. This resulted not only in potential isolation, but different types of “punishments.” The experience of being punished combined with a lack of insight into why they evoked such strong feelings in others, was painful and difficult for many. Ella, 23 years old, describes this:

I played with dolls from when I was very young, and when mum and dad got annoyed with me, or put cars or action figures or Lego in front of me, I started to cry. So, this was a long time before I really started to express myself verbally, so I have felt this since I was very young. But I did not know what it was at that time, right, I did not know what transsexualism is, it is not something you know, you just feel that something is wrong.

The desire to do what is “normal” and “right” combined with a strong belief in what is common and what is unusual, is described by many. Talking about being encouraged or told to play with toys that are defined as “the right” for the opposite sex made the participants confused, and at times even angry. It seemed incomprehensible and unfair to be “punished” for doing something they thought was fun.

Many reflected on the coping strategies they had used to handle bullying and disruptive behavior. Some talked about how they had eaten their lunch in the lavatory, cried in the school restroom, and tried to stay alone but were nonetheless bothered by disrespectful words and behaviors. They describe how they have let bad words hail, pretended not to be hurt, and according to Alexander, 21 years; in the aftermath “*cried more than the amount of fluid in the body indicates is possible*.” Most of the participants underlined that they had never felt “accepted,” and some described previous thoughts about themselves using adjectives like “dirty” and “contagious.”

### The experience of being different and lonely

Most individuals desire a sense of community, fellowship, and belonging to the herd, yet many labeled this as “unthinkable” and “utopian.” Not being invited to birthdays, not being invited home after school, and not having someone to accompany them, to and from school, affected many. Physical alterations when puberty gradually started and developed formed even greater discrepancy in many. Memories where they compared their bodies to other people's bodies induced feelings of frustration and again shame. Negative emotions and intensified feelings of alienation were outlined. Difficulties participating in the gym, swimming and other physical activities made the exclusion even greater and many of the participants describe how the sensation of loneliness grew. The physical changes associated with puberty are described by Ella, 23 years old, who at that time was going through a boy's puberty:

*When I started to become a teenager, or when I started to get a little older, I was around 8–9 years old, and then came those gym classes. Then I remember that the girls started getting such small hormonal breasts then, such small pellets. And the girls compared breast size. ‘Oh, look at me, I have bigger than you,' and then finally I started to stand with my arms a little like crossing over my chest and thought: ‘Where are mine? Why do I not have boobs'*

The fear of physical attributes exposing that they were biologically born the opposite sex, posed a great threat to many and gave rise to a lot of stress in childhood and adolescence. The participants talked about social restrictions they imposed on themselves, such as isolating themselves rather than being with others, and many did not talk to anyone about the feelings that prevailed inside. Kim, 39 years old, talks about the desire to fit in and having a close friend:

I noticed that it was a bit painful in a way not to have ... Just like someone like that, a friend or something like that. Because I did not fit in, so even though they wanted to play with me, it was just like something in me made me not want to, I was not quite like them.

All the participants tell in their own way about how they felt alone in a world where their body did not develop on the outside as they felt on the inside; and how this reinforced the negative spiral related to the feeling of alienation.

The fear that someone would see the genitals in which did not correspond to the identity; thus “revealing” that they are not who they claim to be, were described as overriding and repeated by most participants several times in the interviews. Ingrid, 36 years old, describes this fear in an illustrative way:

I remember very well that there were a lot of such grotesque stories and things that were taken up during parent meetings and stuff, where it was boys and girls in the class who had joined each other in the bathroom and started studying each other, because they have to find out what is the difference between girls and boys, and then I started thinking: What if someone comes and asks me? If they can look at me? And then these thoughts and questions were there about ... This couldn't be right, this does not feel like it's mine, that I should not have it (male genitals). And that was when I somehow first started to feel the feeling of being unhappy as a genetically born male, boy.

Practical challenges such as mandatory activities of changing clothes also created difficult situations and emotions for many in childhood and adolescence. Agnethe, 25 years old, describes how the discomfort exposing her body and genitals meant that she often avoided changing clothes:

Eh. I have a very bad gym grade, so I, because my time in high school was horrible when it came to the gym, eh wardrobe, wardrobe change and such, was some of the worst that existed. So, I simply refused to change gym clothes from time to time in which pulled down my grade a lot so I think I have grade 2 or 3 (one being the lowest) in gymnastics so I think I should just take up the gym again.

Preventing or physically avoiding situations that potentially could expose genitals and reveal a conflicted identity was described as a repetitive pattern that frequently required a management strategy. Loneliness and negative emotions and thoughts are recurring topics in the participants' stories. Three of the participants described the importance of having fantasy friends. However, they expressed that they felt embarrassed and ashamed when their parents' overheard conversations with their fantasy friends and asked whom they were talking to. Gina, 24 years old, tells how she at one point thought that she might be “crazy”:

And then I started to get very, very lonely, and got a very bad thought inside my head that I think about to this day, and it was that I started to think I was crazy. Because I had fantasy friends, because I was a very lonely child.

The fact that the friends only represented a fantasy and not physical friends made many feel that something was wrong with them. This was related to the fact that they did not experience that there were others who had friends who theoretically did not exist. At the same time, these friends were very much alive; they could see them clearly and they represented sources to limit the feeling of loneliness.

### Strategies to protect own identity

The participants' stories about how they expressed themselves in childhood are considered a significant finding. The participants emphasize that in encounters with others, they started to recognize that they were different and divergent from others. Such experiences sheds light on the driving force and motivation behind the person who chooses to take the long journey toward surgically changing their genitals. Several of the participants talked about concealment stories they created in which reflected the time and the world they lived in to become a part of the norm. Similarly, several told how they in their childhood developed strategies for acquiring “girl toys” or “boy toys” and playing with them in secret. Gina, 24 years old, is one of those who describes this:

I had a small wooden box that was out in our doll's house, and I took that wooden box down to the pier where there was a boat, and then I hid that wooden box, under the pier. Had my Barbie dolls hidden there. Every single day in the summer I ran down there, and played for maybe half an hour, 20 minutes, then I had to get back up so that my mother and father were not worried, and then go down again for half an hour, 20 minutes, and play.

Gina told that she made the decision to keep her sentiments and toy preferences a secret out of feelings of embarrassment about not meeting what she believed to be her parents' expectations. Such stories are illustrative of all the participants in the study, and many tell how important it was that others were not to see through what they were really thinking and who they really were. Maria, 21 years old, tells how she deliberately isolated herself both to avoid being exposed to other people's thoughts about her, and to avoid revealing her difficult thoughts about herself:

I go into myself and isolate myself and, and avoid things when I have felt that ... When I have felt anxiety in relation to what people think about me, and what I have between my legs and things like that…

Isolation to avoid “revealing oneself” or “exposing oneself” to the reactions of others was repeatedly described as a type of coping strategy. Another type of strategy was to ensure careful planning of activities that could potentially reveal the side of the identity that were to be kept hidden. Ella, 23 years old, talks about careful planning to ensure that she could play the way she wanted without anyone discovering it:

And then when I was 10–11–12 I had savings, so I took a backpack with me, and inside it I had a hoodie. Then I cycled down to the toy store, bought myself the Disney Barbie dolls, asked if I could borrow the toilet at the toy store, changed my hoodie and cycled home. Because I was so afraid that someone would have seen me there, both friends, family, if there was someone who could tell on me, that I had been to the toy store…

The constant effort of finding ways to make sure that their gender identity was not made visible to the outside world, thus living it out in secret, was one of the most common patterns among the participants. Most of the participants use the classification boys' toys, girls' toys, and the same gender division about clothes. They explicate the discrepancy between what they themselves want and what expectations everyone around them has. The discrepancy seems to be related to the sociocultural norms that govern the surrounding society and how parents' and others' mind-sets influence participants' ideas of what clothes to wear, or should wear, and what is the right way to move and behave when you have either female or male genitals.

To constantly feel that you do not master or want what the environment requires encourages negative emotions that are repeated and reinforced. Negative and conflicting feelings are also linked to the experience that you are constantly lying to others; thus, you present yourself as a different person than you really are. Different coping strategies to hide physical changes associated with puberty are outlined among most of the participants. Some of the measures were simple, such as wearing larger clothes to hide breasts, while other measures caused physical discomfort in the long run, such as choosing to walk with a crooked back even though the musculoskeletal system was affected. Aleksander, 21 years old, talks about such a strategy:

I got my period quite late. And so ... I generally feel that I developed quite late. But when that development first began, I was like this … uff… I did not like it. And when I started getting breasts, I sank down quite a bit, because I did not want it to appear that I had breasts, so I started walking very crooked, and with very baggy clothes.

Keeping alleged gender identity vs. biological gender secret was particularly important for the participants in puberty and adolescence. Three of the participants stated that they did not talk to anyone about how they felt. Agnethe, 25 years old, explains why it was important not to convey her thoughts to anyone, but also that she lacked words to describe what she felt:

I did not tell anyone about it then because it was a bit controversial at the time and honestly, I had no words for what I was feeling so I just lived with it. It is very difficult to describe it to someone who has not felt it in a way. It is like such a feeling of error that only got drastically worse and worse throughout puberty.

Several participants expressed that being all alone with such feelings, triggered the need to stay away from other people, not go out, live their lives via the internet and plan all activities to the smallest detail. Many of the participants also told that they had developed mental health problems over time, and received various types of diagnoses, such as anxiety and depression. Constant inner turmoil in relation to who you are, want to be, and the possibility of making radical changes to get better (hormones and/or surgery) seemed to make the participants find it difficult to sort emotions and think clearly.

### Gender identity and the significance of the genitalia

The significance of having a genital organ that reflects experienced gender identity are emphasized during many interviews. Physical and mental discomfort as well as physical pain from having a genital organ which according to the participant should not be there is another aspect. Ingrid, 36 years old, talks about her relationship to her genitals: “*I'm not myself if I still have it down there, right. That penis. [...] Yes, so I must do what's right for me […]. It is to get it done, to get rid of what should not really be there….”*

Challenging and strong feelings related to the genitals represented for many a major motivation to eventually choose to implement radical changes such as hormone therapy and surgery. The perception of not being complete; that there is something that is “not supposed to be there” or something that is “missing” is described by all the participants. The desire to change this, and unpleasant feelings and inner stress associated with the possibility of such a change, are illustrated in the participants stories. Kim, 39 years old, describes how he thinks being “whole” must be related to having a genital organ that corresponds to a perceived gender identity:

Well, I feel like any man in the street. Just that I'm missing one thing, for me, what I'm missing is the last bit. I just want to feel a completely different feeling... I want to go to the toilet (standing) in the woods when I'm out jogging. It's just knowing that, okay, I have something there (a penis) …

Many describe how the discrepancy between their genitals and the one they identify as is perceived as a paramount and exhausting emotion. Some of the participants had told some friends about their biological sex and the process they were going through, while others had only told it to one or two confidants, for example a friend or parent. Thus, even when becoming an adult there were many difficult situations where the discrepancy between the genitals and the identity being played out, risked being revealed. In situations such as being in the woods, at festivals, in public toilets, it becomes visible how urination is performed, and participants told how they continually prepare and take precautions to ensure that no one can see their genitalia or how they stand or sit.

Participants typically used unfavorable words to describe the external genitalia. Many talked of having a type of pre-puberty curiosity about whether they would have a penis, tits, or vagina. When puberty hit and the opposite of what they expected presented itself, it was a kind of anxiety and a feeling of inaccuracy with discomfort that dominated the descriptions. Elliot, 46 years old, describes this feeling of discomfort: “*Disgust. It was like that, ehh … I did not relate to them (breasts). So, it was just something that hung there, that in a way should not be there.”*

Distaste for what “hung” there or “stood out” is representative of the descriptions of external gender characteristics that did not match. Aids such as tight vests were used to hide the breast for others but mostly for themselves, so they did not trigger bad vibes, in which was somewhat partly successful. Several said that such aids were painful and tight and that taking them on and off triggered the feeling of discomfort. Elliot, goes on to describe this:

You kind of don't get rid of that little indication of breasts. So, I remember when I had my breasts removed, so ... It was almost like that ‘now it can stop here' (the changes) […]. Because it was so liberating.

Agnethe, 25 years old, describes the same feeling of discomfort associated with the genitalia:

The fact that it is there (the penis), seemingly can feel that it is there all the time, it is, it is a bad feeling. Not as bad now as it was before. Now, I can't get a boner (erection) and such things because the hormones reduce it drastically unless you want it yourself. I like…., I have not used it. I use it minimally. Preferably avoid touching it and doing such things.

As described by Agnethe, the feeling of discomfort causes many of the participants to experience cleansing, urination, and sexual stimulation as forms of touch that they may have great difficulty performing or experiencing. Similarly, challenging feelings were portrayed in situations where the genitals were to be exposed or touched by others, as in gynecological examinations: Elliot, 46 years old, describes such a situation: “*It was my second gynecological examination throughout my 35 years, at that time. Ehh, maybe I was 39 years. Yes ... And, it was also like, like just, it was absolutely awful.”*

Lying in a gynecological chair or pulling down your pants while others examine the genitals is by several of the participants described as a demanding feeling that evokes responses described as “nausea,” “disgust,” and “fear.” According to the descriptions depicted by several participants, there are periods when they forget that the outer genitalia do not correspond to their identity and suddenly it is a mirror, a look, a feeling that throws them back into the emotion of discomfort and nausea. Rejecting a body part by not looking at it, not touching it or relating to it in any way, gives participants time and space to forget the feeling of “being wrong.” But as soon as they are reminded of the body part either by themselves, by an emotion, or by a visual reminder, they express the return of hurtful and difficult feelings. Such reminders were often linked to urination and the use of public toilets, but not at least to the inevitable daily exposures and touches. Agnethe, 25 years old, describes the feeling of washing her penis: “*I have to wash my penis because it is hygienic, but it's not exactly something I like to do. I breathe heavily and make grimaces with my face….”*

Having sexual intercourse with others represented a situation in which ambivalent and challenging feelings were activated. Maria, 21 years old, talks about situations where such feelings are evoked:

I look at myself in the mirror, and I shower, and … during sex too, so it can be, that I sometimes feel the sensation ... (bad bodily sensation). Then I interrupt because I suddenly do not feel for it anymore, and it is like ... (pause). When I did not have a regular sex partner, I was more like that, I did not expect them to touch me or things like that. Like not acknowledging it (the penis).

Several of the participants discuss how their age, security, and apparently reliable partners allow them to forget about their discomfort until instances like the ones described above remind them of it. The perception that something is wrong and uncomfortable therefore endures. Several of the participants reported physical and mental changes after commencing hormone therapy. They explain that it causes less discomfort in “being oneself,” but that when difficult and painful emotions arise, it is perceived as very challenging since they have had a break from such experiences.

## Discussion

This study explores patients' thoughts, feelings, and experiences, during childhood and adolescence, as they gradually prepare for genital surgery. Despite individual differences, there are some major themes that should be discussed to better understand why people want to undergo hormone therapy and genital surgery. One of the themes relates to experiences and various cultural conventions of gender expression, which is strongly connected to the meaning of genitalia and gender identity. Another theme relates to the felt need of concealing one's gender identity and body as a form of self-defense, and thus crucial to resolve. This feeling is connected to the experience of being different and not knowing why, and the associated experience of being stigmatized and punished for this difference. Another significant issue concerns the importance of having a genital organ that expresses one's actual gender identity as well as the psychological and physical distress associated with a genital organ that, according to the participant, should not exist or is absent.

According to Norwegian historian and gender researcher Sandal (2017), there has been a considerable change in our understanding of gender and gender identity since the 1950s (2017). Depending on the age of participants, they describe contemporary thoughts about what constitutes a man and a woman in terms of appropriate clothes, mannerisms, and mindset. Thus, it is relevant to contextualize the findings by looking at how the understanding of gender and gender identity is continuously changing, as it has always done historically. Butler (1990) and Foucault (2006) shed light on how the understanding of gender and sexuality, violations of gender norms, and what is considered normal variation or pathology, have changed throughout history—depending on which views dominate. Butler (1990) claimed that although there are physical differences between men and women, you are not born a “woman”; rather, you become one by your actions or performances, actions and performances that are influenced by religion and myths. Further, the role of patriarchy has been strengthened by political structures like inheritance and marriage, and social expectations on how woman are to behave and “perform.” This includes how women are to dress in different phases of life and serves as a form of subjugation that continues into old age. Butler (1990) used Beauvoir's ideas from the book Beauvoir (2015) to support her performativity theories, and according to Beauvoir, becoming a woman is a process that starts in childhood, and as girls enter adolescence, they become more “woman” transforming further into the “other sex.” Sexuality and sexual initiation represent the final stages of entering the role of the woman, while motherhood completes the girls transition to “woman.” Even though the concept of gender and gender identity has altered dramatically, one might discuss to what extent socio-cultural expectations and imaginations still shape the perceptions of what constitutes a man and a woman—and how those expectations affect the individuals' perception of themselves. A systematic review of studies carried out globally reveals that gender variant people face greater rates of violence and discrimination in job, healthcare, and housing across cultures as a population traditionally punished for violating gender norms (Reisner et al., 2016). According to Goffman (1990), the basis of stigmatization processes consists of comparable mechanisms of assessments based on normative expectations of how a person should behave in society. If it is concluded that a person expresses or performs in deviant ways (such as a girl wearing masculine clothes) or has “attributes” that are inconsistent with the characteristics of the category of people to which they belong (such as a boy with breasts), these inconsistencies devalue them as undesirable and flawed, potentially designating them as members of a stigmatized group (Goffman, 1990, p. 12). Those that are stigmatized normally come from the same cultural background as their appraisers. As a result, the individual will be conscious of the fact that they fall short of the social norm of the group to which they strive. This self-stigma may have negative effects on the person's conception of themselves and cause shame (Goffman, 1990, p. 18). In other words, stigma and shame may be elements that foster the emergence of psychological suffering. To put this concern in context, we must recognize that gender is both a biological feature and a socially and culturally controlled term (Bussey and Bandura, 1999). Boys and girls recognize their differences early on, since adults treat them differently regardless of situation, and youngsters respond appropriately. Depending on which society life is played out in, and to a greater or lesser extent depending on that society, there are different written and unwritten rules for how girls and boys, women and men, should behave. They are expected to perform different roles and duties, and they face different expectations in terms of characteristics and capabilities (von Tetzchner, 2018). Children's gender awareness involves both real conditions, such as the fact that boys grow up to be men and girls grow up to be women, and society's expectations of how boys and girls behave in various situations (Von Tetzchner, 2001). Several interviewees stated that they had known from childhood that what they were doing or how they were “performing” (Goffman, 1956) did not conform to established, culturally specific norms. Thus, expressing oneself in contradiction to such norms caused a self-inflicted shame.

Our study shows that to prevent being identified as their opposite ascribed gender, many act and “perform” in line with what is (assumingly) expected. Goffman's (1956) “dramaturgical model of social interaction” illustrates how a person continually negotiates their image and appearance in interactions with other people to fit into a norm. By moving between backstage and frontstage, playing out various roles, such as “forbidden” gender-related activities backstage, the participants managed to entertain their perceived gender and at the same time (to a certain extent) avoid stigmatization when being exposed to the public eye.

Trying to avoid being exposed, colored the childhood and adolescents among the participants. This specific behavioral pattern of playing out different parts of the identity in different “stages” could be conscious or partly subconscious. Most people acknowledge that those ranked low in the social order, such as stigmatized groups, frequently face daily injustice or “small miseries,” illuminated by Bourdieu's (1999, 4) idea of social suffering. Because of how society devalues difference and the stigma attached to it, human difference can be experienced not only as small miseries but also represent a barrier to lifetime functioning (Kittay, 2006). In the study by Jessen et al. (2021) on subjective GD experiences among help-seeking transgender and gender non-confirming youth, participants described emotional recollections of feeling different and left out among peers as a child. Some linked their sentiments to their incongruent gender, while others linked them to a general sense of not belonging and that these emotional experiences still tormented them. That is consistent with the experiences of many of the participants in our study who recalled attempting to conform to culturally accepted behavior in the name of what were seen as “natural,” often resulting in inconsistent behavior and humiliating situations.

Goffmann (1992) outlines how we categorize others and how others classify us, as well as how we react to others because of these classifications. It can be interpreted to mean that we can't relate to one another without placing each other in categories. Our study shows that some children as young as 4–5 years old employed covert tactics, deception, and making up stories while interacting with adults. In this way they could perform according to the anticipated roles of their biological gender “frontstage” (Goffmann, 1992), while living out their perceived identity on the covert “backstage.” However, living with these inconsistencies, and having to change in between “stages” or categories, was described as influential in the chosen group's decisions regarding initiating hormone therapy, and finally surgery. All the participants, both those who had been accepted by their families and communities, and those who had not, expressed a strong and consistent desire for genital surgery. It is, however, difficult to say, were the feelings of being “wrong” derives from. Traditional gender roles, gender-specific toys, clothes, looks, behavior, and body language have all been highlighted by multiple participants as major factors in feeling “wrong.” Thus, societal and cultural trends seem to have a strong influence and feed the idea of being born in the wrong body. Furthermore, social medias allow people to interact with like-minded people in support groups, enhancing acceptance of who they are.

In the study by Jessen et al. (2021), most of the participants stated that their subjective experiences with gender dysphoria were impacted by their interactions with others. They had also experienced that forming bonds and relationships with people could help relate to their bodies in new ways, decreasing their subjective symptoms of gender dysphoria. In our study, we found that such bonds seemed to increase the individual's self-esteem, and thus the stages might “flip.” In other words, some participants started to live out their self-identified gender frontstage, even before initiating hormonal treatment or surgical operations. Some experienced that they no longer felt the need to conceal or act, and with the right support and acceptance from others, the symptoms of gender dysphoria could modify, but also sometimes persist. One of the findings of the study by Jessen et al. (2021) was that living as the identified gender had been a transforming experience that had affected how participants saw their history. The participants expressed relief, stating that they now could understand their former sentiments and emotional recollections of being different in a another perspective. This is consistent with many of our participants' accounts, who told that they were able to relax more and weren't always defending who they were after they began to recognize and behave somewhat in accordance with their perceived genders. However, the findings also show that, dependent on the situation, participants were constantly attempting to somehow regulate the impressions (impression management) (Goffman, 1956). The notion that someone would see that their genitalia did not correlate to who they claimed to be in society, was characterized as an immensely scary thought, and can be interpreted as feelings strongly encouraging the need for an exterior alteration such as surgery. In the study by Jessen et al. (2021) some participants told that despite their best efforts; they were still unable to reduce subjective gender dysphoria experiences and thus struggled with shame, guilt, and confusion.

In our study we found that even though several of our participants underwent socially inspired alterations, they all experienced dysphoria in the sense of distaste for their genitals. Further, they all continued to require surgery even if friends, family, and their community accepted their gender identity. The findings describing distaste and the experience of being stigmatized, both felt and enacted, seem to be related (Goffman, 1956). External body parts were frequently described in negative ways by many participants. Many remembered a sense of wonderment about what they believed would happen when they reached puberty. When puberty arrived and the opposite of what they expected occurred, descriptions of discomfort, anxiousness, and a sense of inaccuracy predominated. Some also described how people seemed to be staring at them, and that some acted aggressively toward them. This type of enacted stigma, combined with general socio-cultural expectations, may cause or re-enforce self-stigma as people internalize these public attitudes and suffer numerous negative consequences as a result (Corrigan and Rao, 2012).

All the study participants emphasized the significance of having a genital organ that corresponded to their gender identity. Through their narratives we learned that the physical and mental pain of having a genital organ that did not suit their identity grew, and this sensation served as a constant reminder of not being complete; that there was something “missing” or something that should not have been there. Rejecting a body part by not looking at it, not touching it or relating to it in any way, was a way of handling feelings of “being wrong.” However, as soon as they were reminded of the body part either by themselves, by others, or by an emotion, the difficult feelings returned. In their study, Jessen et al. (2021) mentions a theme called bodily sensations, which refers to numerous types of difficult bodily experiences that occur during the day and defines “embodied pain” as the anguish and dysphoria that persons who go through “wrong” puberty experience. The study by Jentoft (2019) study showed how entering the “wrong puberty” often resulted in impression control measures such as wearing clothing (binders) over newly formed body parts like breasts. This is consistent with our participants' accounts of growing pains, genital discomfort, and continuous reminders during bathroom visits, showering, sexual intimacy or intercourse, and other everyday activities, culminating in several concealment strategies, avoidance of activities, and sometimes development of social anxiety. As emphasized earlier, international studies find that gender variant young persons may be more likely to struggle with depression, suicidal ideology, anxiety, eating disorders, and self-harm compared to cisgender peers (Grossman and D'Augelli, 2007; Grossman et al., 2011; Connolly et al., 2016; Olson et al., 2016).

In a study that examined whether distress and impairment, two essential traits of mental disorders, could be explained by social rejection and violent experiences rather than being characteristics of transgender identity, it was discovered that social rejection and violence were highly predictive of distress and all types of dysfunctions (Robles et al., 2016). LGBTQ groups are often affected by what is termed minority stress, which is caused by sexism toward men and women who express their gender, identify, or sexuality differently from the norm and who are criticized or derided for their appearance or behavior (López-Sáez et al., 2020). According to Winter et al. (2016) health disparities among transgender people, particularly in terms of mental health, are like a slope that leads from stigma to illness. Thus, the stigma transgender people encounter throughout their lives can be a major contributor to the disparities in mental health and wellbeing that persist also after transition and/or care that is gender affirming (Robles et al., 2016; Winter et al., 2016). Up until 2019, the WHO's diagnosis manual classified transgender persons as having mental disorders, which shaped how transgender people received healthcare; likely reinforced stigma; and likely had a significant negative influence on their health and welfare (World Health Organization, 2019). Thus, stigma and social suffering may be factors that create conditions for psychological suffering to emerge (Robles et al., 2016), and should be included as a perspective when discussing and developing health policies for transgender people. As suggested by Winter et al. (2016) governments and other public entities should invest in public education about gender incongruence so that transgender people can experience full social inclusion. Additionally, those who work with transgender persons, such as health care professionals, need to be trained to deliver services that are responsive to the needs and rights of transgender people. Such efforts may not only reduce self-inflicted stigma but also enhance resilience to societal stigma and social suffering among transgender people.

### Weaknesses and strength of the study

The study has relatively few informants and including more participants could have added more perspectives. Another weakness could be that the interviewer has worked with the patient group for 10 years, thus more difficult to maintain an open mind during analysis. However, the second author has no experience with the topic, and the analysis and the results are based on discussions and negotiations between the two authors. All the participants had early onset gender incongruence, and all had undergone genital surgery. Thus, the participants had many experiences in common and they had all taken the radical choice of going through surgery. Understanding the background for this choice was the main focus of this part of the study. Another strength was that the participants were interviewed both before and after surgery, and in this way we were able to elaborate several of the topics addressed in the first interview. Another strength is the obvious public interest in this topic and the study mad add new knowledge and understanding that may inspire the already ongoing public debates.

## Future research

We suggest that future studies focus on quality of life after genital surgery, and within this focus address topics as sexual function after genital surgery, including potential pains and complications. Further, it would be interesting to explore different dimensions of sexuality, both before and after genital surgery, and also how social function and issues related to mental health may change after surgery.

## Data availability statement

The raw data supporting the conclusions of this article will be made available by the authors, with some reservations.

## Ethics statement

The studies involving human participants were reviewed and approved by the Regional Committee for Medical and Health Research Ethics (REK), which gave its prior approval (2019/731 B). The patients/participants provided their written informed consent to participate in this study.

## Author contributions

LB and MS were responsible for the conception of the study, development of the study design, and drafting the manuscript. LB conducted the data collection, while both authors participated in the data analysis. All authors have read and approved the final manuscript and are accountable for all aspects of the work.
